# Hemophilia Severity and Its Association With Mental Health and Health‐Related Quality of Life—Results From a Cross‐Sectional Multicenter Study

**DOI:** 10.1111/hae.70219

**Published:** 2026-01-30

**Authors:** Francesca Schmitt, Laura Maier, Stefan Lerch, Manuela Albisetti, Alice Trinchero, Lukas Graf, Heinz Hengartner, Pierre Fontana, Nicolas von der Weid, Katrin Scheinemann, Sylvia von Mackensen, Corinna Reichl, Marialuisa Cavelti, Ines Mürner‐Lavanchy, Johanna A. Kremer Hovinga, Michael Kaess, Mutlu Kartal‐Kaess

**Affiliations:** ^1^ Division of Pediatric Hematology & Oncology Department of Pediatrics Inselspital University Hospital University of Bern Bern Switzerland; ^2^ University Hospital of Child and Adolescent Psychiatry and Psychotherapy University of Bern Bern Switzerland; ^3^ Graduate School for Health Sciences University of Bern Bern Switzerland; ^4^ Division of Pediatric Hematology University Children`s Hospital Zurich Zurich Switzerland; ^5^ Department of Medical Oncology and Hematology University Hospital Zurich Zurich Switzerland; ^6^ Center For Laboratory Medicine and Hemophilia Hemostasis Center St. Gallen Switzerland; ^7^ Division of Oncology/ Hematology Children's Hospital of Eastern Switzerland St. Gallen Switzerland; ^8^ Division of Angiology and Hemostasis University Hospitals of Geneva Geneva Switzerland; ^9^ Division of Pediatric Hematology and Oncology University Children's Hospital Basel University of Basel Basel Switzerland; ^10^ Faculty of Health Sciences and Medicine University Lucerne Lucerne Switzerland; ^11^ Department of Medical Psychology University Medical Center Hamburg‐Eppendorf Hamburg Germany; ^12^ Faculty of Psychology University of Basel Basel Switzerland; ^13^ Department For BioMedical Research University of Bern Bern Switzerland; ^14^ Department of Hematology and Central Hematology Laboratory Inselspital Bern University Hospital University of Bern Bern Switzerland; ^15^ Department of Child and Adolescent Psychiatry Center For Psychosocial Medicine University Hospital Heidelberg Heidelberg Germany

**Keywords:** health‐related quality of life, hemophilia, mental disorders, mental health, psychopathology

## Abstract

**Background:**

Limited existing research on mental health and health‐related quality of life (HRQoL) in people with hemophilia (PwH) suggests these patients still may have poor mental health despite treatment advances significantly improving somatic outcomes.

**Objectives:**

This multicenter study aimed to systematically assess mental health and HRQoL and their association with disease severity, age and treatment regimen among PwH.

**Methods:**

This cross‐sectional study, conducted in nine Swiss hemophilia treatment centers, included participants aged six years and older with congenital hemophilia of any severity. The study procedure comprised a semi‐structured psychiatric diagnostic interview and an online survey comprising age‐validated psychological measures to capture mental health and HRQoL. Treatment centers provided clinical data.

**Results:**

Of 164 PwH enrolled in the study, 156 participants completed the psychiatric diagnostic interview. 25% met the criteria for a mental disorder (MD). Most common among the MD were affective disorders, substance use disorders, and attention deficit hyperactive disorder. Moderate/severe hemophilia and lower baseline factor activity were significantly associated with higher psychopathology and lower HRQoL. Of participants with moderate/severe hemophilia, 26% of those on prophylaxis versus 45% of those on on‐demand met the criteria for an MD.

**Conclusions:**

Elevated prevalence of MD, and the association of psychopathology with disease severity and treatment regimen, highlights the continued relevance of mental health in hemophilia research. Further objective clinical research is indispensable to define targets for improved and individualized comprehensive treatment care plans.

**Plain Language Summary:**

Major treatment advances have transformed hemophilia care, allowing most people with hemophilia (PwH) to have average lifespans. Reduced bleeding complications have shifted attention to overall well‐being and mental health of PwH. The multicenter Swiss HERMES study examined 164 children and adults with hemophilia to explore the link between disease severity, treatment regimen, mental health, and health‐related quality of life using standard questionnaires and a psychiatric interview. About one in four participants had at least one mental disorder—most often depression, substance use disorders, or ADHD. Participants with moderate or severe hemophilia, or lower clotting factor levels, reported poorer health‐related quality of life. Prophylactic treatment may support mental health in participants with moderate or severe hemophilia. These findings show that mental health issues are common in PwH and highlight the need to integrate psychological screening and support into comprehensive hemophilia care.

## 1 Introduction

Advances in hemophilia treatment over the past 50 years have transformed interdisciplinary clinical management and focus [1, 2]. New therapies are typically evaluated by their efficacy in reducing annualized bleeding rates (ABR) and somatic comorbidities. With treated people with hemophilia (PwH) now achieving near‐normal life expectancy, attention has shifted towards outcomes beyond ABR and joint health to capture the broader impact of emerging therapies [3]. Despite improvement in clinical symptoms, health‐related quality of life (HRQoL) remains compromised in PwH [4]. Studies have shown that PwH report lower HRQoL compared to the general population [5]. Research on adolescents and young adults with bleeding disorders links chronic pain to worse physical and mental HRQoL, as measured by generic HRQoL tools [6].

While generic HRQoL measures (g‐HRQoL) enable comparisons between PwH, the general population, and other chronic conditions, hemophilia‐specific HRQoL measures (hs‐HRQoL) offer deeper insights into disease‐related factors [7]. Elderly PwH report lower hs‐HRQoL—particularly in physical health, activity and leisure—often reflecting a poor orthopedic status [8]. Prior research also found associations between HRQoL and disease severity [9, 10]. Conversely, prophylaxis and extended half‐life (EHL) products have been linked to HRQoL improvements, potentially due to reduced bleeding, higher factor activity [11, 12, 13], and psychosocial benefits such as less parental overprotection compared with on‐demand therapy [14]. Age remains critical in evaluating HRQoL. Children and adolescents might deviate in HRQoL patterns from adults due to developmental differences [14]. Older PwH often experience cumulative comorbidities and infections (e.g. hepatitis C and human immunodeficiency virus (HIV)), given that viral inactivation of plasma‐derived factor replacements was first introduced in 1985 and prophylactic care in the 1990s [15].

A key contributor to reduced HRQoL in PwH may be a high prevalence of mental health problems [5], which can impair treatment adherence and overall well‐being [16]. To date, mental health remains underexplored in hemophilia research. For instance, a multicenter study of 1,400 PwH (including 417 children) across 21 European countries assessed HRQoL but not psychopathology [17]. Existing studies on mental disorders (MD) in PwH are limited, focusing on specific subpopulations or psychopathological symptoms. In adult Portuguese PwH, anxiety and depression symptoms were reported in 36.3% and 27.5%, respectively, and were associated with more hospital visits, bleeding episodes, affected joints, pain, and poorer HRQoL [18]. A Nordic study found depression or anxiety due to hemophilia (28%) increased with disease severity, but only assessed PwH on SHL products [9]. Pediatric research has mainly examined ADHD in PwH, with limited data on other MD [19].

Understanding the prevalence and impact of MD in PwH is impeded by prior studies with inconsistent findings, small or unrepresentative samples, and assessment methods varying reliability [9, 20, 21, 22]. Moreover, evolving hemophilia treatments and changing rates of HIV and hepatitis may have influenced MD prevalence, underscoring the need for updated data.

HERMES (HEmophilia‐Related MEntal health and illnesS) is a cross‐sectional, observational, multicenter study in Switzerland designed to clarify associations between hemophilia and mental health. Employing standardized, age‐specific psychometric interviews and questionnaires in a high‐resource setting, HERMES examines MD prevalence, dimensional psychopathology, HRQoL, and their relationship to disease severity. It further explores whether these associations differ by age or treatment regimen.

## 2 Methods

### 2.1 Study Design and Participants

HERMES is a cross‐sectional study conducted between November 2020 and January 2023 in nine hemophilia treatment centers (HTC) across six Swiss cities (Bern, Zürich, St. Gallen, Geneva, Basel, Aarau; see Appendix).

Included were male patients aged ≥6 years with congenital hemophilia A (HA) or B (HB) of any severity receiving standard care [23]. Exclusion criteria included acquired hemophilia or insufficient German/French proficiency. For minors (<18 years old), parents participated; written informed consent was obtained from all participants or parents (for those <14 years).

### 2.2 Study Procedure

Participants completed a structured psychiatric interview, psychopathology and HRQoL questionnaires, and a demographic/medical history survey. Missing clinical data were supplemented by HTC staff. Diagnostic interviews were conducted by trained psychologists or physicians via telephone or video call, with independent inter‐rater reliability checks. For children under 12, parents completed the interview by video with the child present, then parents provided answers for questionnaires reporting on the child within the same appointment. Questionnaires assessing parent reports on themselves were sent out afterwards. Older participants (≥12 years) completed interviews by phone. The online questionnaires (survey) were sent for participants afterwards to complete at their own pace. If any questionnaires remained incomplete after two weeks, study staff followed up with a reminder. All study materials were available in German or French. Participants received a compensation (gift voucher worth CHF 50) after completing the survey.

An emergency plan was in place in case acute suicidal thoughts or plans were communicated by participants during the interview. Immediate reporting by study staff, consulting a local psychiatrist and referral to the nearest local psychiatry of the participant was defined. For each participating study center a local psychiatric center in the canton was defined to be contacted in case of emergency.

### 2.3 Demographic and Hemophilia‐Related Characteristics

Participants provided sociodemographic (age, ethnicity [Caucasian or white/Asian/African/Mixed/Other]), and clinical characteristics (hemophilia type [HA/HB], lowest baseline factor activity ever measured [%], severity [mild: >5–<40%, moderate: 1–5%, severe: <1%] [23], previous inhibitor [Yes/No], HIV [Yes/No], hepatitis [Yes/No], family history of hemophilia [Yes/No], treatment regimen [on‐demand/prophylaxis], product category [SHL/EHL/NFT/None/Other], annual bleeding rate [number of bleeding episodes per year], target joint [Yes/No/Unknown], and any pain, acute or chronic, in the last 3 months [Yes/No]). For analysis, hemophilia severity was grouped as mild hemophilia and moderate/severe hemophilia.

### 2.4 Mental Health

**Mini‐International Neuropsychiatric Interview (M.I.N.I.)**— The M.I.N.I. (version 6.0) [24] and its child version (M.I.N.I.‐Kid) [25] assessed DSM‐5 [26] and ICD‐10 [27] mental disorders.

**Symptom Checklist‐90‐Revised (SCL‐90‐R)**— The 90‐item SCL‐90‐R measured psychological distress in adults [28]. Total scores were transformed (0–1), with higher values indicating higher psychopathology.

**Strengths and Difficulties Questionnaire (SDQ)**— The 25‐item SDQ assessed emotional and behavioral difficulties in participants aged 4–17 years [29]. Parents of children <12 completed proxy reports; older participants self‐reported. Scores were transformed (0–1), with higher scores reflecting greater difficulties.

### 2.5 Hemophilia‐Specific Health‐Related Quality of Life (hs‐HRQoL)

**Haemophilia‐Specific Quality of Life Assessment Instruments (Haemo‐QoL I/II/III, Haem‐A‐QoL)**— Parents of participants aged 6–11 years completed proxy versions (Haemo‐QoL I, 29 items; Haemo‐QoL II,64 items), adolescents aged 12–17 years self‐reported (Haemo‐QoL III, 84 items) [7], and adults used the Haem‐A‐QoL (91 items) [30]. Scores were linearly transformed and reversed (−1 to 0) so that higher scores represented better hs‐HRQoL to match the direction of the g‐HRQoL scores, with higher scores indicating better hs‐HRQoL.

### 2.6 Generic HRQoL (g‐HRQoL)

**Health‐Related Quality of Life Questionnaire for Children and Adolescents (Kiddy‐, Kid‐, Kiddo‐KINDL)**— Parents of participants <12 years provided proxy reports using the Kiddy‐ or Kid‐KINDL (24 items each); adolescents (12–17 years) completed age‐appropriate self‐reports (Kid‐ or Kiddo‐KINDL; 24 items each) [31, 32]. Total scores were transformed (0–1), with higher values indicating better g‐HRQoL.

**The 36‐Item Short Form Health Survey (SF‐36)**— Adults completed the 36‐item SF‐36 [33], a validated g‐HRQoL measure widely used in hemophilia and other chronic illnesses [34]. Total scores were transformed (0–1) for analysis with higher scores indicating better g‐HRQoL.

### 2.7 Statistical Analysis

Analyses were conducted using STATA version 18.5 Standard Edition (Stata College Station, Texas). Categorical data are presented as frequencies and percentages. Continuous data are presented as means (*M*) and standard deviations (*SD*) as well as medians (*Mdn*) and interquartile ranges (*IQR*). Age was standardized to the sample mean: z‐scores (*z*). Questionnaire total scores were linearly transformed (range: 0–1) to merge comparable constructs to form variables with data across participants of all ages (severity of psychopathology, hs‐HRQoL and g‐HRQoL). Hs‐HRQoL transformed scores were then reversed (range: ‐1–0) to align directions with g‐HRQoL scores. Only interviews and questionnaires with no missing items were analyzed.

Logistic regressions tested whether hemophilia severity, baseline factor activity, or treatment regimen predicted meeting criteria for a MD. Linear regressions tested whether severity of hemophilia, baseline factor activity or treatment regimen predicted continuous outcome variables (severity of psychopathology, hs‐HRQoL, g‐HRQoL). Age was tested as a moderator. Previous inhibitors and pain in the last 3 months were tested as possible confounding factors. Logistic regressions are reported as odds ratios (*OR*). Linear regressions are reported as unstandardized beta coefficients (*β*). Statistical significance was set at *p* < 0.05; however, all P‐values and 95% confidence intervals (CI) are reported. As residuals deviated from normality (Shapiro–Wilk *p* < 0.05), robust standard errors were applied.

## 3 Results

### 3.1 Sample Characteristics

**FIGURE 1:**
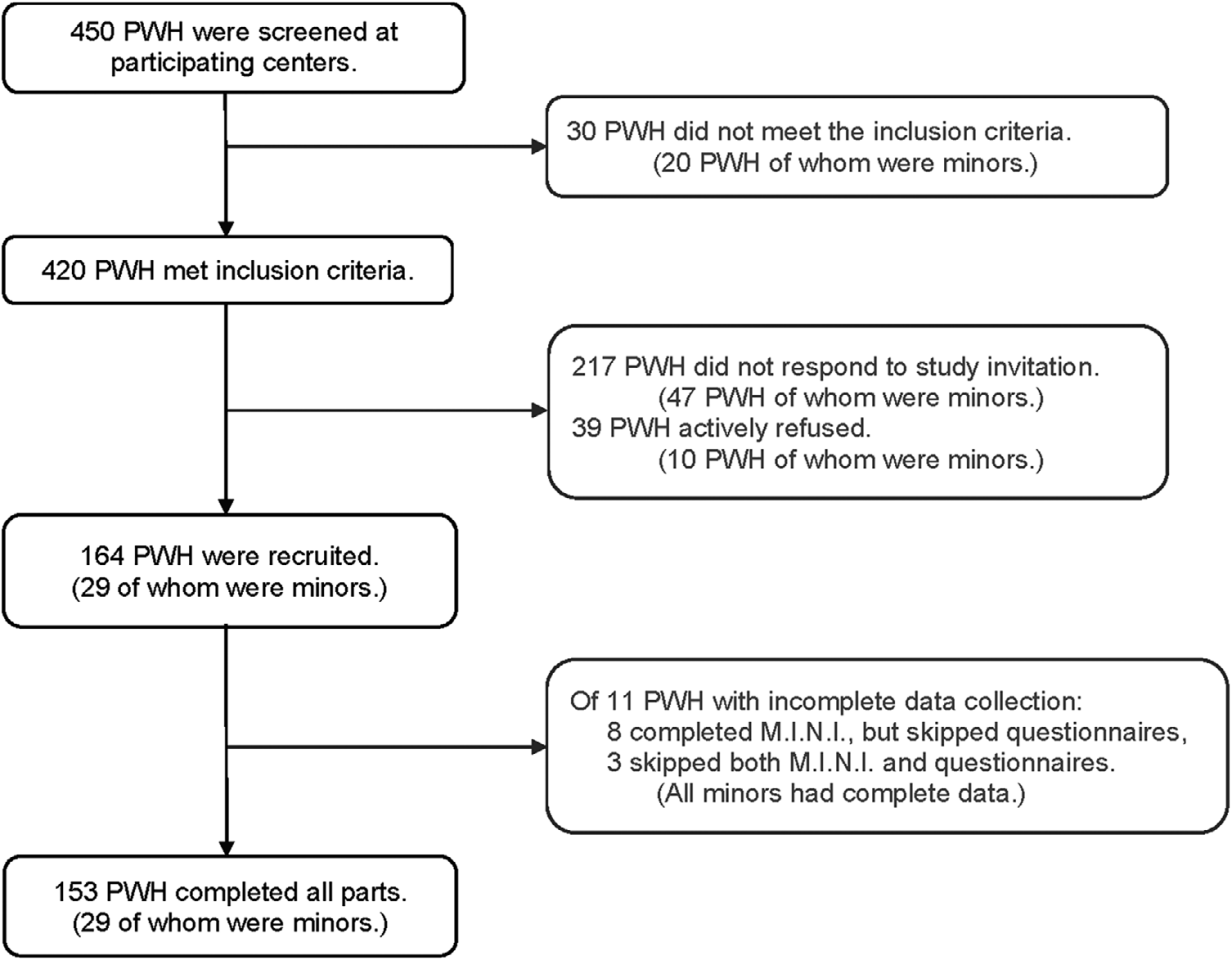
HERMES study recruitment flowchart. M.I.N.I., mini‐international neuropsychiatric interview; minors, participants < 18 years of age; PwH, people with hemophilia.

Of 420 eligible PwH, 164 (39%) signed informed consent. Medical data were available for all 164 participants. 153 (93%) completed the questionnaires and the psychiatric diagnostic interview. A further three participants only partook in the psychiatric diagnostic interview (Figure 1).

Participants had a mean age of 38.9 (*SD* = 20.0, *Mdn* = 37, *IQR* = 32, range: 6–84) years. The sample included 29 minors, whose parents (1 father and 28 mothers) contributed to the assessment. Of all participants, 134 (82%) had hemophilia A (HA), and 30 (18%) had hemophilia B (HB). 85 (52%) participants had severe, 32 (19%) had moderate, and 47 (29%) had mild hemophilia. 97 (59%) participants were on prophylactic treatment, while 67 (41%) were on an on‐demand treatment. Further characteristics are reported in Table 1. Characteristics for participants on prophylaxis are summarized by product category (SHL, EHL, NFT) in Supplemental Table 1. Data on previous inhibitors, pain in the last 3 months and their association with mental health and HRQoL outcomes are reported in Supplemental Table 3.The impact of treatment regimen (on‐demand vs. prophylaxis) on mental health and HRQoL in participants with moderate/severe hemophilia (*n* = 117) was analyzed on 113 (97%) participants with complete data.

**TABLE 1:** Clinical characteristics of PwH.

		Hemophilia severity		Treatment regimen	
	Whole sample	Mild	Moderate/severe	Prophylaxis	On‐demand
*n* (%^a^)	164 (100)	47 (29)	117 (71)	96 (59)^b^	67 (41)
Age in years, *M (SD)*	38.9 (20.0)	42.6 (21.7)	37.5 (19.2)	36.2 (19.3)	43.2 (20.4)
**Ethnicity, *n (%)***					
Caucasian/white	155 (95)	45 (96)	110 (94)	90 (94)	64 (97)
Asian	4 (2)	0 (0)	4 (3)	3 (3)	1 (1)
African	0 (0)	0 (0)	0 (0)	0 (0)	0 (0)
Mixed	4 (2)	1 (2)	3 (3)	3 (3)	1 (1)
Other	1 (1)	1 (2)	0 (0)	0 (0)	1 (1)
**Type of hemophilia, *n (%)***					
Hemophilia A	134 (82)	37 (79)	97 (83)	79 (82)	54 (81)
Hemophilia B	30 (18)	10 (21)	20 (17)	17 (18)	13 (19)
Baseline factor activity in percent, *M (SD)*	5.5 (9.1)	16.9 (9.6)	0.8 (1.5)	0.5 (1.2)	12.7 (10.6)
**Treatment regimen, *n (%)***					
Prophylaxis	97 (59)	1 (2)	96 (82)	96 (100)	—
On‐demand	67 (41)	46 (98)	21 (18)	—	67 (100)
**Product category, *n (%)***					
SHL	58 (36)	33 (70)	25 (21)	12 (13)	46 (69)
EHL	84 (51)	9 (19)	75 (64)	68 (71)	16 (24)
NFT	17 (10)	1 (2)	16 (14)	16 (16)	4 (6)
None/other	5 (3)	4 (9)	1 (1)	0 (0)	1 (1)
**Target joint, *n (%)***					
Yes	36 (22)	1 (2)	35 (30)	32 (33)	4 (6)
No	126 (77)	45 (96)	81 (69)	63 (66)	62 (93)
Unknown/not reported	2 (1)	1 (2)	1 (1)	1 (1)	1 (1)
Bleeds per year, *M (SD)*	2.4 (4.3)	0.9 (1.5)	3.1 (4.9)	3.2 (5.2)	1.4 (2.3)
**Pain in last 3 months, *n (%)***					
Yes	89 (54)	17 (36)	72 (62)	64 (67)	24 (36)
No	63 (38)	23 (49)	40 (34)	28 (29)	35 (52)
Unknown/not reported	12 (8)	7 (15)	5 (4)	4 (4)	8 (12)
**Previous inhibitor, *n (%)***					
Yes	17 (10)	3 (6)	14 (12)	11 (11)	5 (7)
No	147 (90)	44 (94)	103 (88)	85 (89)	62 (93)
HIV, *n (%)*					
Yes	4 (2)	0 (0)	4 (3)	4 (4)	0 (0)
No	160 (98)	47 (100)	113 (97)	92 (96)	67 (100)
**Hepatitis, *n (%)***					
Yes	3 (2)	0 (0)	3 (3)	3 (3)	0 (0)
No	161 (98)	47 (100)	114 (97)	93 (97)	67 (100)
**Positive family history, *n (%)***					
Yes	75 (46)	21 (45)	54 (46)	43 (45)	32 (48)
No	83 (50)	22 (47)	61 (52)	51 (53)	31 (46)
Unknown/not reported	6 (4)	4 (8)	2 (2)	2 (2)	4 (6)

Abbreviations: EHL, extended half‐life; HIV, human immunodeficiency virus; *n*, number of patients; NFT, non‐factor replacement therapy; PwH, people with hemophilia; SHL, standard half‐life.

^a^ Percentages in this row are calculated with respect to the whole sample (*N* = 164).

^b^ One participant with mild hemophilia A was on prophylactic treatment. He was excluded from the table to avoid skewing in for example, baseline factor activity.

### 3.2 Mental Health

A total of 39 out of 156 (25%) participants met criteria for a MD over their lifetime, and 15 (10%) met criteria for more than one MD. The most common MD were affective disorders and substance use disorders (Supplemental Table 2). Prior to the diagnostic interview, 138 (88%) participants did not report a MD in the general clinical assessment; however, 24 (21%) of these participants met criteria for at least one MD in the diagnostic interview. Based strictly on the diagnostic interview, 4 of 29 (14%) children met criteria for an ADHD diagnosis. Five further participants reported having a doctor‐diagnosed ADHD, although their symptoms did not meet threshold for a M.I.N.I.‐diagnosed ADHD. All but one of these five participants reported taking prescribed pharmacological ADHD treatment to neutralize their symptoms.

**FIGURE 2:**
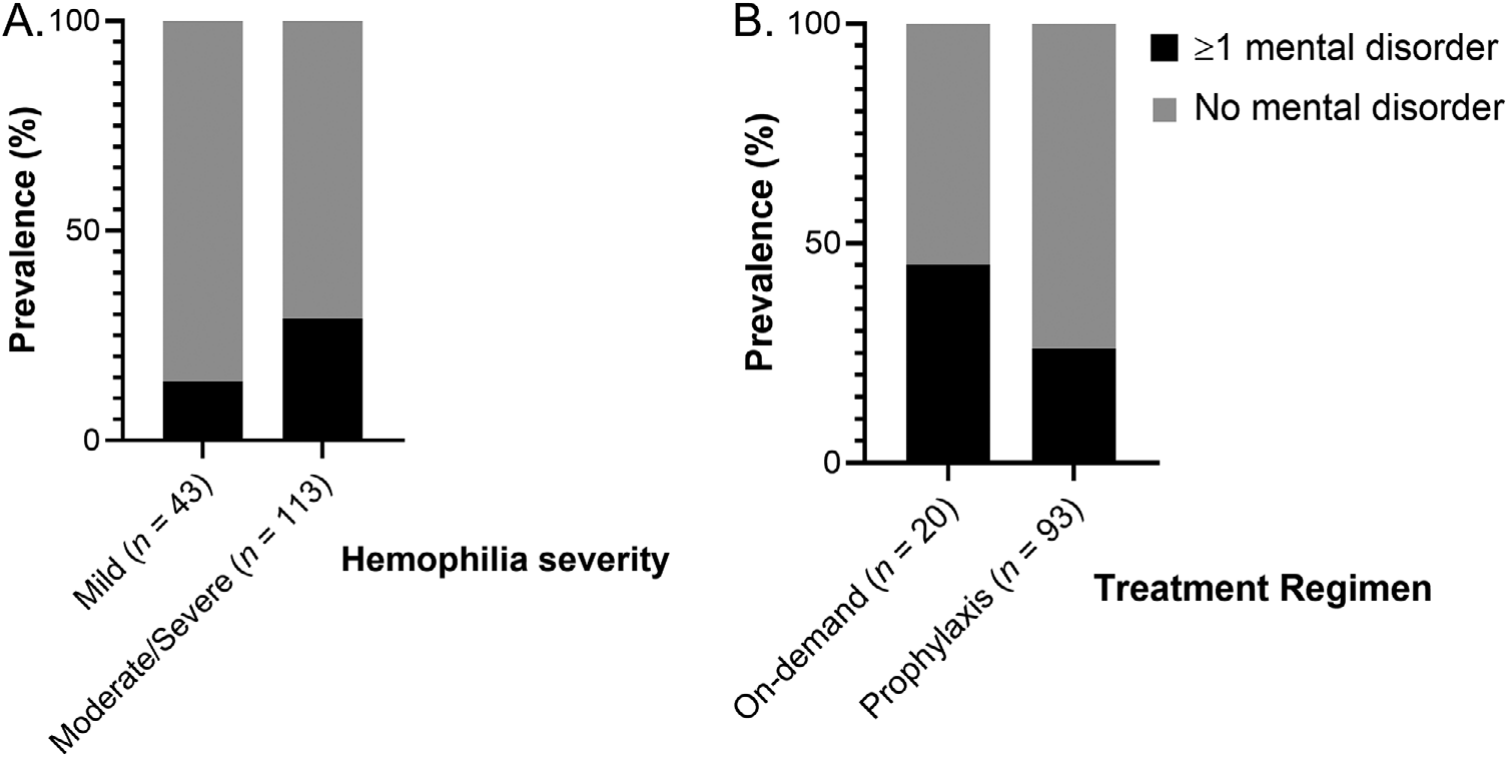
Prevalence of M.I.N.I.‐diagnosed mental disorders across subgroups. Depicted are bar graphs visualizing the prevalence of mental health disorders in 156 participants who underwent the mini‐international neuropsychiatric interview (M.I.N.I.), according to A) hemophilia severity B) and treatment regimen. Black bars represent the percentage of participants who met criteria for a mental disorder; grey represents the percentage who did not meet criteria for a mental disorder.

The prevalence of MD was 29% in participants with moderate/severe hemophilia compared to 14% in those with mild hemophilia (Figure 2A). Participants with moderate/severe hemophilia showed a trend‐significance towards higher likelihood to meet criteria for a MD (*OR* = 2.544, *95% CI* [0.981, 6.598], *p* = 0.055, data not shown). Baseline factor activity did not predict meeting criteria for a MD (*OR* = 0.960, *95% Cl* [0.913, 1.010], *p* = 0.114, data not shown).

**FIGURE 3:**
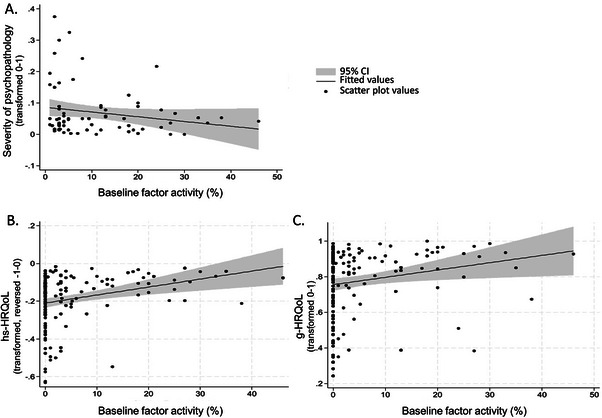
Outcome scores and baseline factor activity. The panel of plots display questionnaire scores for A) severity of psychopathology, B) hemophilia‐specific health‐related quality of life (hs‐HRQoL) and C) generic health‐related quality of life (g‐HRQoL) with respect to baseline factor activity. Black dots represent individual values; black lines represent the fitted values; the grey shading represents the 95% confidence interval (CI).

Severity of psychopathology scores are summarized by hemophilia severity in Table 2. Moderate/severe hemophilia was associated with higher severity of psychopathology (Table 2). Higher baseline factor activity significantly predicted lower psychopathology scores (*β* = −0.002, *95% CI* [−0.003, −0.001], *p* = 0.002; Figure 3A). Addition of previous inhibitor or pain in the last 3 months as control variable did not change results (Supplemental Table 3).

**TABLE 2:** Outcome summary across subsamples.

	Hemophilia severity (*n* = 153)					Treatment regimen (*n* = 113)				
	Mild (*n* = 40)	Moderate/Severe (*n* = 113)	*β*	*95% CI*	*P*	On‐demand (*n* = 20)	Prophylaxis (*n* = 93)	*β*	95% *CI*	*P*
	*M (SD)*	*M (SD)*				*M (SD)*	*M (SD)*			
Construct	*Mdn (IQR)*	*Mdn (IQR)*				*Mdn (IQR)*	*Mdn (IQR)*			
Psychopathology	0.060 (0.071)	0.090 (0.102)	0.031	[0.002, 0.060]	0.039^*^	0.081 (0.111)	0.092 (0.101)	0.011	[−0.042, 0.064]	0.682
	0.042 (0.064)	0.044 (0.133)				0.035 (0.049)	0.047 (0.136)			
hs‐HRQoL	−0.121 (0.092)	−0.211 (0.142)	−0.091	[−0.130, −0.052]	<0.001^***^	−0.192 (0.163)	−0.215 (0.138)	−0.023	[−0.100, 0.053]	0.551
	−0.010 (0.092)	−0.169 (0.163)				−0.215 (‐0.195)	−0.174 (0.163)			
g‐HRQoL	0.849 (0.149)	0.751 (0.195)	−0.098	[−0.157, −0.039]	0.001^**^	0.751 (0.237)	0.751 (0.186)	−0.001	[−0.111, 0.110]	0.993
	0.906 (0.119)	0.823 (0.269)				0.871 (0.290)	0.803 (0.270)			

Abbreviations: β, unstandardized beta coefficient; CI, confidence intervals; g‐HRQoL, generic health‐related quality of life; hs‐HRQoL, hemophilia‐specific health‐related quality of life; IQR, interquartile range; M, mean; Mdn, median; n, number of participants; P, P‐value; SD, standard deviation from the mean.

Outcomes are summarized for the samples used in the regression analysis. 153 participants had complete questionnaire data. Regression analysis assessing treatment regimen included only participants with moderate/severe hemophilia who had complete questionnaire data (*n* = 113). Asterisks indicate significance levels

* *P* < 0.05, ** *P* < 0.01, *** *P* < 0.001.

### 3.3 Health‐Related Quality of Life

HRQoL scores are summarized by hemophilia severity in Table 2. Participants with moderate/severe hemophilia reported lower hs‐HRQoL and g‐HRQoL than participants with mild hemophilia (Table 2). Baseline factor activity was positively associated with both hs‐ and g‐HRQoL scores (hs‐HRQoL: *β* = 0.004, *95% CI* [0.003, 0.006], *p* < 0.001; g‐HRQoL: *β* = 0.004, *95% CI* [0.001, 0.007], *p* = 0.008; Figure 3B&C). Addition of previous inhibitor or pain in the last 3 months as control variable did not change results (Supplemental Table 3).

### 3.4 Role of Age

**FIGURE 4:**
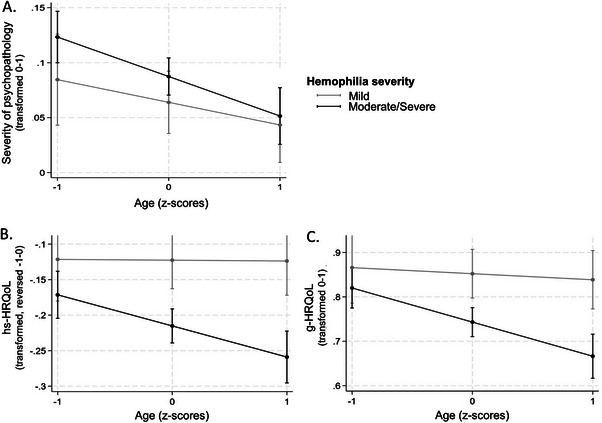
Age effects on psychopathology and HRQoL. Depicted are A) severity of psychopathology, B) hemophilia‐specific health‐related quality of life (hs‐HRQoL), and C) generic health‐related quality of life (g‐HRQoL) scores of each hemophilia severity (mild, grey line; moderate/severe, black line) observed across participants’ ages (*z* ^−1^ = 18.8 years, *z* ^0^ = 38.9 years, *z* ^1^ = .59.0 years). Error bars visualize the 95% confidence intervals for these predicted values.

Age neither moderated the relationship between hemophilia severity and MD (*OR* = 1.429, *95% CI* [0.568, 3.594], *p* = 0.449; data not shown), nor between hemophilia severity and severity of psychopathology (*β* = ‐0.015, *95% CI* [−0.046, 0.015], *p* = 0.320; Figure 4A). Age moderated the association between hemophilia severity and HRQoL (hs‐HRQoL: *β* = −0.044, *95% CI* [−0.085, −0.002], *p* = 0.039; g‐HRQoL: *β* = −0.061, *95% CI* [−0.120, −0.003], *p* = 0.041). While age did not affect HRQoL scores in participants with mild hemophilia, participants with moderate/severe hemophilia showed decreasing HRQoL scores with increasing age (Figure 4B&C). Age did not moderate the relationships between baseline factor activity and MD or HRQoL (MD: *OR* = 0.997, *95% CI* [0.948, 1.048], *p* = 0.895; hs‐HRQoL: *β* = 0.001, *95% CI* [−0.000, 0.003], *p* = 0.153; g‐HRQoL: *β* = 0.001, *95% CI* [−0.001, 0.004], *p* = 0.262; data not shown). Age showed a positive moderation of the relationship between baseline factor activity and severity of psychopathology (*β* = 0.001, *95% CI* [0.000, 0.003], *p* = 0.014; data not shown).

### 3.5 Role of Treatment Regimen

The analysis examining the impact of treatment regimen (on‐demand vs. prophylaxis) on mental health and HRQoL in participants with moderate/severe hemophilia (*n* = 117) was conducted on 113 (97%) participants with complete data. 20 of those participants injected on‐demand, and 93 injected prophylactically. 15 of the 20 participants on on‐demand treatment, and 16 of the 93 participants on prophylaxis had moderate hemophilia. The prevalence of MD was 26% in participants treating prophylactically compared to 45% in among the participants treating on‐demand (Figure 2B). Severity of psychopathology and HRQoL total scores are reported by treatment regimen in Table 2.

Treatment regimen did not predict prevalence of MD (MD: *OR* = 0.425, *95% CI* [0.157, 1.151], *p* = 0.092; data not shown), severity of psychopathology, hs‐HRQoL or g‐HRQoL (Table 2).

## 4 Discussion

This cross‐sectional, multicenter study enrolled 164 children, adolescents, and adults with hemophilia of varying severities; 93% had complete data. One‐fourth met criteria for at least one MD, and 10% for more than one. The most common MD were affective disorders, substance use disorders, and ADHD. Moderate/severe hemophilia and lower baseline factor activity were significantly associated with higher psychopathology and lower HRQoL. Age amplified the negative association between HRQoL and hemophilia severity and buffered the negative link between psychopathology and baseline factor activity. Among participants with moderate/severe hemophilia, 26% on prophylaxis versus 45% on on‐demand therapy met criteria for a MD, though treatment regimen was not significantly associated with outcomes.

Adult PwH had a higher prevalence of MD than adults of the general Swiss population (27% vs. 18%) [35]. Affective and substance use disorders predominated, consistent with previous reports [36]. Previous studies report anxiety/depression symptoms (not diagnoses) in 27–36% of adults with hemophilia [9]. Affective disorders in our adult sample (10%) were comparable to rates in diabetes [37]. Comparing affective symptoms to affective disorders explains why our prevalence is lower than previous hemophilia reports. ADHD prevalence among minors (13.8%) exceeded global prevalence [38] and aligns with prior findings in boys with hemophilia [19, 22]. We only report prevalence of ADHD in minor participants, as ADHD is not in the M.I.N.I. version for adults. Only MD meeting M.I.N.I.(‐Kid) criteria were included to foster reproducibility of mental health reports in preceding hemophilia literature [20]. Recall bias may have influenced lifetime reports. As some participants on psychiatric medications did not meet diagnostic criteria for a MD at assessment, MD prevalence is likely an underestimation.

MD were more frequent in moderate/severe than mild hemophilia (29% vs. 14%). Participants with moderate/severe disease had approximately 2.5‐fold higher odds of lifetime MD, showing trend significance (*p* = 0.055). Similarly, lower baseline factor activity predicted higher psychopathology scores. These findings support prior evidence of affective disorders across all severities but with higher prevalence in more severe disease [9, 20].

Participants with moderate/severe hemophilia reported worse hs‐HRQoL and g‐HRQoL than those with mild disease. These associations were confirmed in regression analyses using baseline factor activity. Age moderated HRQoL: older participants with moderate/severe hemophilia had poorer HRQoL, while mild cases showed no age effect—in concordance with prior findings [9, 10, 11, 12, 14]. Older PwH may experience compounded comorbidities due to later prophylaxis initiation, whereas younger generations may benefit from early modern therapies [15]. HRQoL trajectories are expected to further improve with novel treatments.

Age did not moderate the relationship between MD and hemophilia severity. Contrary to expectations, older PwH reported lower psychopathology severity, mirroring trends in the general population where mental health improves with age [35]. Given mental health's influence on treatment adherence [16], early psychosocial support may be key to optimizing outcomes, particularly for young PwH facing treatment and social challenges [12]. Participants on prophylaxis tended towards lower MD prevalence, but treatment regimen did not significantly predict mental health or HRQoL outcomes. As most on‐demand patients had hemophilia, collinearity between disease severity and treatment likely biased results. Prior work highlights prophylaxis benefits for moderate hemophilia, improving long‐term somatic and HRQoL outcomes [13]. Our findings indirectly support revisiting treatment guidelines for this group.

A major strength of HERMES is the assessment of MD is the use of standardized diagnostic interviews by trained clinicians, minimizing subjectivity compared to questionnaire‐only studies [9, 20, 21, 22]. Inclusion across two major Swiss language regions enhances representativeness, and consistent care within the national network limits center‐based variation. Standardized measures were employed to support comparison across ages and (healthcare) settings. Transforming questionnaire scores afforded age‐appropriate analysis across a span of ages. Participants received care at centers affiliated within the Swiss Hemophilia Network, thus differences in care between centers are supposed to be minimal.

Limitations include exclusion of children under six, reducing insight into early psychosocial challenges. The extension of the study over two language regions within the country (German and French) depicted the majority of Switzerland, but may be limited in its representation of PwH of other Swiss language regions. Findings may generalize mainly to high‐resource settings. As data collection occurred soon after NFT introduction in Switzerland, most participants were treated with EHL products; future HRQoL may differ with the increasing use of NFT, ultra‐long‐acting factor concentrates and gene therapy. The cross‐sectional design precludes causal inference or longitudinal assessment. Modest recruitment (39% of eligible patients over 20 months) and lack of data on non‐participants may have introduced bias. Participation bias could affect our results in either direction: PwH with severe mental health burden may have not had the capacity to participate, or PwH with mental health concerns felt drawn to participate. Furthermore, we note a comparably low prevalence of previous inhibitors (10%) in our study population. Future analyses should further investigate associations of clinical characteristics such as past and present inhibitors, (chronic) pain and MD/HRQoL. Though not a focus of this manuscript, our data could not report on current inhibitors, nor distinguish whether pain experienced in the last 3 months was chronic or acute. These variables may be of interest in future studies of mental health and HRQoL in PwH.

We found that MD are overrepresented in PwH and are more prevalent in patients with moderate/severe hemophilia, particularly when treating on‐demand. These findings highlight the need to integrate standardized mental health screening into interdisciplinary care in HTCs. The high prevalence of ADHD found in children with hemophilia merits further investigation. Future research of mental health and HRQoL in PwH will need to be longitudinal to track long‐term trajectories of PwH receiving modern treatments. Objective clinical research is indispensable to ascertain the burden of MD in PwH and inform individualized, comprehensive treatment strategies in the era of gene and novel therapies.

## Author Contributions

MKK, JAKH and MK conceived and designed the study. FS, LM, SL conducted primary analyses of the data. FS, SL, CR, MC, IML, MK, MKK performed further analyses and/or participated in data interpretation. MA, AT, LG, HH, PF, NW, KS, JAKH, MKK performed collection of clinical data. SvM provided the hs‐HRQoL questionnaires. FS, MC and MKK wrote the draft of the manuscript. FS, MC and MKK drafted figures. All authors revised the manuscript and approved the final version for submission.The funding source had no role in the study design, data collection, data analysis, data interpretation or writing of the report. All authors confirm that they had full access to all the data in the study and accept responsibility to submit for publication.

## Funding

The authors have nothing to report.

## Ethics Statement

The study was approved by all relevant ethics committees (lead: Kantonale Ethikkommission Bern, KEK‐ID: 2020‐01945), and complied with the Declaration of Helsinki [39] and Good Clinical Practice [40]. A STROBE checklist can be found in the Supplement.

## Conflicts of Interest

FS, LM, SL, NW, KS, CR, MC, IML, and MK have nothing to declare during the past 3 years related to the present work.

MA reports travel support from SOBI and NovoNordisk.

AT reports consulting/advisory role: NovoNordisk, Pfizer, Roche, SOBI, Takeda; research funding: Octapharma, NovoNordisk; travel, accommodations, expenses: NovoNordisk, SOBI.

LG reports consulting/advisory role: CSL Behring, NovoNordisk, Pfizer, Roche, SOBI; funding: Roche, SOBI; travel, accommodations, expenses: NovoNordisk, SOBI.

HH reports travel support from SOBI and participation on the SOBI Advisory Board.

PF reports travel support from SOBI and NovoNordisk and advisory fees from Roche.

SvM declares consultancy: Roche, Spark, Pfizer; Research Funding: Biomarin, SOBI, Takeda; Speakers Bureau: Biomarin, Takeda, Chugai, Kedrion; Membership of an entity's Board of Directors or advisory committee: Biomarin, Chugai/Roche.

JAKH reports consulting/advisory role and/or lecture fees from Ablynx/Sanofi, Bayer, Roche, SOBI, and Takeda. All honoraria go to the employer, Insel Gruppe AG. In addition, she received congress travel support from Roche, SOBI, and Takeda.

MKK reports honoraria paid to the institution (consultancy, speaker, chair, educational events): Roche, SOBI, Takeda, Pfizer, NovoNordisk; fees to the institution for study participation: Roche, Pfizer, SOBI, NovoNordisk; travel support from: NovoNordisk, SOBI, Takeda.

## Declaration of Generative AI and AI‐Assisted Technologies in the Writing Process

The authors used OpenAI. (2025). *ChatGPT* [Large language model]. https://chatgpt.com/ in the revision stage of the writing process to meet word count requirements.

## Supporting information




**Supplemental Table 1**: Clinical characteristics and outcomes of participants on prophylaxis.
PwH, people with hemophilia; SHL, standard half‐life; EHL, extended half‐life; NFT, non‐factor replacement therapy; g‐HRQoL, generic health‐related quality of life; HIV, Human immunodeficiency virus; hs‐HRQoL, hemophilia‐specific health‐related quality of life; MD, mental disorder.
^ a^ *n* = 93 of 96 participants on prophylaxis completed the psychiatric interview.
^ b^ *n* = 93 completed the questionnaires.

**Supplemental Table 2**: Summary of detected MD across the whole sample.
Detected mental health disorders are categorized according to the ICD‐10 classification [27].
^ a^ Percentages in this column are calculated with respect to the n = 156 patients, who completed the diagnostic interview.

**Supplemental Table 3**. Outcomes stratified by previous inhibitors and pain
^ a^ Results in this column are calculated with respect to the n = 156 patients, who completed the diagnostic interview.
^ b^ Results in this column are calculated with respect to the n = 153 patients, who completed the questionnaires.

Supporting File 4: hae70219‐sup‐0004‐SuppMat.docx

## Data Availability

The data presented are available from the authors with the permission of the involved local investigators.

## Participating Swiss Hemophilia Treatment Centers

### 1

M Kartal‐Kaess, Division of Pediatric Hematology & Oncology, Department of Pediatrics, Inselspital, University Hospital Bern

JA Kremer Hovinga, Department of Hematology and Central Hematology Laboratory, Inselspital, University Hospital Bern

M Albisetti, Pediatric Hematology, University Children`s Hospital Zurich

A Trinchero, Department of Medical Oncology and Hematology, USZ, University Hospital Zurich

L Graf, Center for Laboratory Medicine, Haemophiliezentrum St. Gallen

H Hengartner, Division of Oncology/ Hematology, Children's Hospital of Eastern Switzerland, St. Gallen

P Fontana, HUG Unité d'Hémostase Hémophilie adulte, Genève

N von der Weid, Pediatric Hematology and Oncology, Department of Pediatrics, University of Basel, UKBB

K Scheinemann, Division of Oncology/ Hematology, Department of Pediatrics, Kantonsspital Aarau, Kinderspital Aarau (until November 2022)
