# Supplementary material for: Hemophilia Severity and Its Association With Mental Health and Health‐Related Quality of Life—Results From a Cross‐Sectional Multicenter Study
Source: Haemophilia. 2026 Jan 30;32(3):748–59. doi: 10.1111/hae.70219 (PMC13175438; doi:10.1111/hae.70219)
Supplement: Supplementary file 1 — Supplemental Table 1: Clinical characteristics and outcomes of participants on prophylaxis. PwH, people with hemophilia; SHL, standard half‐life; EHL, extended half‐life; NFT, non‐factor replacement therapy; g‐HRQoL, generic health‐related quality of life; HIV, Human immunodeficiency virus; hs‐HRQoL, hemophilia‐specific health‐related quality of life; MD, mental disorder. a n = 93 of 96 participants on prophylaxis completed the psychiatric interview. b n = 93 completed the questionnaires. [file HAE-32-748-s003.docx]

|  | Product category | | | Total |
| --- | --- | --- | --- | --- |
|  | *SHL* | *EHL* | *NFT* |  |
| Group size, *n (%)* | 12 (13) | 68 (71) | 16 (16) | 96 (100) |
| Age (years, *M ± SD*) | 28.4 ± 9.7 | 36.8 ± 20.7 | 39.7 ± 17.6 | 36.2 ± 19.3 |
| Ethnicity, *n (%)* |  |  |  |  |
| Caucasian/white | 12 (100) | 63 (93) | 15 (94) | 90 (94) |
| Asian | 0 (0) | 3 (4) | 0 (0) | 3 (3) |
| African | 0 (0) | 0 (0) | 0 (0) | 0 (0) |
| Mixed | 0 (0) | 2 (3) | 1 (6) | 3 (3) |
| Other | 0 (0) | 0 (0) | 0 (0) | 0 (0) |
| Type of hemophilia, *n (%)* |  |  |  |  |
| Hemophilia A | 11 (92) | 52 (76) | 16 (100) | 79 (82) |
| Hemophilia B | 1 (8) | 16 (24) | 0 (0) | 17 (18) |
| Baseline factor activity, (%, *M ± SD)* | .17 ± .58 | .47 ± 1.1 | .47 ± 1.2 | .43 ± 1.1 |
| ≥1 Target joint, *n (%)* |  |  |  |  |
| Yes | 0 (0) | 25 (37) | 7 (44) | 32 (33) |
| No | 11 (92) | 43 (63) | 9 (56) | 63 (66) |
| Unknown/not reported | 1 (8) | 0 (0) | 0 (0) | 1 (1) |
| No. bleeds/year, (*M ± SD)* | 1.3 ± 1.4 | 3.6 ± 5.3 | 3.0 ± 6.4 | 3.2 ± 5.2 |
| Pain in last 3 months, *n (%)* |  |  |  |  |
| Yes | 6 (50) | 47 (69) | 11 (69) | 64 (67) |
| No | 5 (42) | 18 (26) | 5 (31) | 28 (29) |
| Unknown/not reported | 1 (8) | 3 (4) | 0 (0) | 4 (4) |
| Previous inhibitor, *n (%)* |  |  |  |  |
| Yes | 3 (25) | 3 (4) | 5 (31) | 11 (11) |
| No | 9 (75) | 65 (96) | 11 (69) | 85 (89) |
| HIV, *n (%)* |  |  |  |  |
| Yes | 1 (8) | 2 (3) | 1 (6) | 4 (4) |
| No | 11 (92) | 66 (97) | 15 (94) | 92 (96) |
| Hepatitis, *n (%)* |  |  |  |  |
| Yes | 0 (0) | 3 (4) | 0 (0) | 3 (3) |
| No | 12 (100) | 65 (96) | 16 (100) | 93 (97) |
| Positive family history, *n (%)* |  |  |  |  |
| Yes | 7 (58) | 30 (44) | 6 (38) | 43 (45) |
| No | 4 (33) | 37 (54) | 10 (62) | 51 (53) |
| Unknown/not reported | 1 (9) | 1 (2) | 0 (0) | 2 (2) |
| Outcome measures^a, b^ | | | | |
| MD *≥1*, *n (%)^a^* | 2 (19) | 20 (30) | 2 (13) | 24 (26) |
| Psychopathology, (*M* ± *SD)^b^* | 0.03 ± 0.04 | 0.11 ± 0.15 | 0.07 ± 0.09 | 0.09 ± 0.10 |
| hs-HRQoL, (*M* ± *SD)^b^* | -0.13 ± 0.10 | -0.24 ± 0.15 | -0.17 ± 0.08 | -0.22 ± 0.14 |
| g-HRQoL, (*M* ± *SD)^b^* | 0.87 ± 0.48 | 0.72 ± 0.19 | 0.78 ± 0.14 | 0.75 ± 0.19 |
