# Supplementary material for: Hemophilia Severity and Its Association With Mental Health and Health‐Related Quality of Life—Results From a Cross‐Sectional Multicenter Study
Source: Haemophilia. 2026 Jan 30;32(3):748–59. doi: 10.1111/hae.70219 (PMC13175438; doi:10.1111/hae.70219)
Supplement: Supplementary file 2 — Supplemental Table 2: Summary of detected MD across the whole sample. Detected mental health disorders are categorized according to the ICD‐10 classification [27]. a Percentages in this column are calculated with respect to the n = 156 patients, who completed the diagnostic interview. [file HAE-32-748-s002.docx]

| Detected mental disorders (MD) | Prevalence, *n (%*^a^*)* |
| --- | --- |
| ≥1 MD | 39 (25) |
| ≥2 MD | 15 (10) |
| F10-19: Psychological and behavioral disorders by psychotropic substances | 15 (10) |
| F20-29: Schizophrenia, schizotypal, delusional, and other non-mood psychotic disorders | 4 (3) |
| F30-39: Affective disorders | 25 (16) |
| F40-49: Neurotic, stress-related and somatoform disorders | 13 (8) |
| F69-69: Disorders of adult personality and behavior | 2 (1) |
| F90-F98: Behavioral and emotional disorders beginning in childhood and adolescence | 4 (3) |
