# Supplementary material for: Hemophilia Severity and Its Association With Mental Health and Health‐Related Quality of Life—Results From a Cross‐Sectional Multicenter Study
Source: Haemophilia. 2026 Jan 30;32(3):748–59. doi: 10.1111/hae.70219 (PMC13175438; doi:10.1111/hae.70219)
Supplement: Supplementary file 3 — Supplemental Table 3. Outcomes stratified by previous inhibitors and pain a Results in this column are calculated with respect to the n = 156 patients, who completed the diagnostic interview. b Results in this column are calculated with respect to the n = 153 patients, who completed the questionnaires. [file HAE-32-748-s004.docx]

|  | Mental health | | | | | | | | | Health-related quality of life | | | | | | | | |
| --- | --- | --- | --- | --- | --- | --- | --- | --- | --- | --- | --- | --- | --- | --- | --- | --- | --- | --- |
|  | Mental disorder*^a^* | | | | | Psychopathology*^b^* | | | | Generic*^b^* | | | | Hemophilia-specific*^b^* | | | | |
|  | No,  *n (%)* | ≥1,  *n (%)* | *OR* | *95% CI* | *P* | *M (SD)* | *β* | *95% CI* | *P* | *M (SD)* | *β* | *95% CI* | *P* | *M (SD)* | *β* | *95% CI* | *P* |  |
| Previous inhibitor |  |  | 1.76 | [0.55, 5.62] | .377 |  | -0.01 | [-0.05, 0.03] | .611 |  | -0.04 | [-0.14, 0.05] | 0.355 |  | -0.00 | [-0.07, 0.06] | .932 |  |
| Yes | 9 (64) | 5 (36) |  |  |  | 0.07 (0.08) |  |  |  | 0.74 (0.17) |  |  |  | -0.19 (0.12) |  |  |  |  |
| No | 108 (76) | 34 (24) |  |  |  | 0.08 (0.10) |  |  |  | 0.78 (0.19) |  |  |  | -0.19 (0.14) |  |  |  |  |
| Pain in last 3 months* |  |  | 3.38 | [1.42, 8.02] | .006 |  | 0.02 | [-0.01, 0.05] | .271 |  | -0.18 | [-0.23, -0.13] | <.001 |  | -0.12 | [-0.16, 0.09] | <.001 |  |
| Yes | 59 (67) | 29 (33) |  |  |  | 0.09 (0.10) |  |  |  | 0.70 (0.20) |  |  |  | -0.24 (0.14) |  |  |  |  |
| No | 55 (87) | 8 (13) |  |  |  | 0.07 (0.10) |  |  |  | 0.88 (0.09) |  |  |  | -0.11 (0.08) |  |  |  |  |
| Unknown/not reported | 3 (60) | 2 (40) |  |  |  | 0.16 (0.00) |  |  |  | 0.74 (0.00) |  |  |  | -0.45 (0.00) |  |  |  |  |
